# *Mir34a* constrains pancreatic carcinogenesis

**DOI:** 10.1038/s41598-020-66561-1

**Published:** 2020-06-15

**Authors:** Ana Hidalgo-Sastre, Clara Lubeseder-Martellato, Thomas Engleitner, Katja Steiger, Suyang Zhong, Judit Desztics, Rupert Öllinger, Roland Rad, Roland M. Schmid, Heiko Hermeking, Jens T. Siveke, Guido von Figura

**Affiliations:** 1 Klinik und Poliklinik für Innere Medizin II, Technical University of Munich, Munich, Germany; 2 Institute of Molecular Oncology and Functional Genomics, Department of Medicine II and TranslaTUM Cancer Center, Klinikum rechts der Isar, Technical University of Munich, Munich, Germany; 3 Institute of Pathology, Technical University of Munich, Munich, Germany; 4 Experimental and Molecular Pathology, Institute of Pathology, Ludwig Maximilian University Munich, Munich, Germany; 5 German Cancer Consortium (DKTK), Partner site Munich, Munich, Germany; 6 Institute for Developmental Cancer Therapeutics, West German Cancer Center, University Hospital Essen, Essen, Germany; 7 Division of Solid Tumor Translational Oncology, German Cancer Consortium (DKTK, partner site Essen) and German Cancer Research Center, DKFZ, Heidelberg, Germany; 8 German Cancer Consortium (DKTK), Partner site Essen, Essen, Germany

**Keywords:** Cancer, Gastroenterology

## Abstract

Several studies have shown that over 70 different microRNAs are aberrantly expressed in pancreatic ductal adenocarcinoma (PDAC), affecting proliferation, apoptosis, metabolism, EMT and metastasis. The most important genetic alterations driving PDAC are a constitutive active mutation of the oncogene *Kras* and loss of function of the tumour suppressor *Tp53* gene. Since the MicroRNA 34a (*Mir34a*) is a direct target of *Tp53* it may critically contribute to the suppression of PDAC. *Mir34a* is epigenetically silenced in numerous cancers, including PDAC, where *Mir34a* down-regulation has been associated with poor patient prognosis. To determine whether *Mir34a* represents a suppressor of PDAC formation we generated an *in vivo* PDAC-mouse model harbouring pancreas-specific loss of *Mir34a* (*Kras*^*G12D*^*; Mir34a*^Δ/Δ^). Histological analysis of *Kras*^*G12D*^*; Mir34a*^Δ/Δ^ mice revealed an accelerated formation of pre-neoplastic lesions and a faster PDAC development, compared to *Kras*^*G12D*^ controls. Here we show that the accelerated phenotype is driven by an early up-regulation of the pro-inflammatory cytokines TNFA and IL6 in normal acinar cells and accompanied by the recruitment of immune cells. Our results imply that *Mir34a* restrains PDAC development by modulating the immune microenvironment of PDAC, thus defining *Mir34a* restauration as a potential therapeutic strategy for inhibition of PDAC development.

## Introduction

Pancreatic cancer is the third-leading cause of cancer-related death in the world, with a 5-year survival rate which, despite the great scientific efforts and new therapeutic technology, has only improved from 5% to 8% in the last years^1^. This low survival rate is the result of a combination of factors, including lack of early symptoms, lack of non-invasive detection methods and/or biomarkers, strong resistance of the tumour to chemotherapy, and a rapid metastatic spread. Surgery is the only curative option, but only a small percentage of patients qualify for resection at the time of diagnosis^2^.

A constitutively active form of the *KRAS* oncogene is the main driver mutation of pancreatic ductal adenocarcinoma (PDAC) occurring in 90% of the cases^3^; out of these, the substitution of G12D occurs in 41% of the cases, followed by G12V occurring in 34% of the cases and G12R in 16% of the cases^4^. *Kras*^*G12D*^ expression in epithelial cells leads to activation of inflammatory pathways and results in paracrine signalling with the surrounding stroma. This promotes formation and maintenance of a desmoplastic, fibro-inflammatory microenvironment, which favours the step-wise progression of normal exocrine pancreas into pre-invasive precursor acinar to ductal metaplasia (ADM) and pancreatic intraepithelial neoplasia (PanIN) lesions and PDAC development^5^. Additionally, loss of function of tumour suppressor genes, such as *p53*, *p16* and *SMAD4*, also drives progression of the disease^6^.

MicroRNAs (miRNAs) are small non-coding RNAs of 20 to 25 nucleotides in length that regulate gene expression at the posttranscriptional level by binding the 3′-untranslated region of target mRNAs suppressing their translation and promoting their degradation^7,8^. Increasing evidence has shown that the expression of miRNAs is deregulated in human cancers affecting hallmark processes, such as proliferation, apoptosis, metabolism, EMT and metastasis^9–11^. Several microRNA encoding genes are induced by p53, among them the miR-34 gene family^12^. The Mir34 family is composed of Mir34a, Mir34b and Mir34c. In humans, MIR34b and c are located in chromosome 11q23.1 and transcribed from the same poly-cistronic transcript (they are located in the same exon), whereas MIR34a is encoded by a separate transcript located on chromosome 1p36.22. While MIR34b/c are mainly expressed in lung tissue^13^, MIR34a is ubiquitously expressed in all tissues^14–16^; suggesting tissue-specific functions for the different members of the Mir34 family (this is also the case in mice^14^). Members of the Mir34 family are directly activated by p53^14,17–19^, among them, *Mir34a* is a well-known key tumour suppressor^15,20,21^. In cancer cells, there are two main mechanisms to inactivate tumour suppressor genes: transcriptional silencing by methylation of CpG islands and genomic loss. Evidence of both mechanisms have been reported for miR-34: previous studies showed how the CpG islands in the *Mir34a* promoter are methylated (correlating with *Mir34a* silencing) in different solid tumours, including pancreatic cancer^16,22^; and, genomic loss of the chromosomal region (1p36) of *Mir34a* has been reported in neuroblastoma^20,23^.

Several studies confirmed that *Mir34a* is downregulated in PDAC and many other cancers (reviewed in^10,24,25^) and that it blocks tumour growth by inhibiting genes involved in various oncogenic signalling pathways. *In vitro* studies revealed that MIR34a is downregulated in human pancreatic cancer cells^26^, where it modulates Notch1 signalling, Bcl2, and EMT^27–31^.

In human PDAC patients, loss of MIR34a expression is associated with poor patient prognosis, and MIR34a levels in serum have been proposed as diagnostic biomarker for PDAC^30,32–34^. Additionally, *Mir34a* has a great therapeutic potential^35^, and it has already been tested in pre-clinical studies, where *Mir34a* mimics (in combination with PLK1 siRNA) was delivered using an amphiphilic nano-carrier and led to improved therapeutic response in mice^36^.

Based on the above data, a tumour suppressive function of *Mir34a* is assumed, however, this has not been functionally tested *in vivo*. Here, we present an *in vivo* study of the tumour suppressive role of *Mir34a* in PDAC using genetically engineered mouse models. *Mir34a* is conditionally inactivated in pancreatic tissue in a Kras^G12D^-driven PDAC model, leading to the fast development of pre-neoplastic lesions and PDAC. This acceleration of the phenotype is driven by a cell-autonomous mechanism whereby the acinar cells generated an autocrine inflammatory response leading to recruitment of immune cells.

## Results

### *Mir34a* knockout mice rapidly develop pancreatic lesions at an early stage

To study the role of *Mir34a* in pancreatic carcinogenesis, the effect of conditionally knocking out *Mir34a* during pancreatic exocrine development was examined by crossing *Mir34a*^*fl/fl*^ mice^37^, with *Ptf1a*^*+/Cre*^ mice (hereafter called: *Mir34a*^*Δ/Δ*^) (Supplementary Fig. 1A). Since *Mir34a* is not expressed in normal pancreas, impairment of pancreatic exocrine development was not expected. As predicted, *Mir34a*^*Δ/Δ*^ mice developed normally and showed no obvious phenotype. Body weight and pancreas body weight ratio were comparable between the two groups (Supplementary Fig. 1B,C), and neither macroscopic nor histological differences were observed (Supplementary Fig. 1D,E). Furthermore, expression of pri-*Mir34a* in *Mir34a*^*Δ/Δ*^ mice was undetectable like on the WT controls (Supplementary Fig. 1F). Additionally, no compensatory effect arising from the expression of the other members of the Mir34 family (namely, *Mir34b* and *Mir34c*), was found (Supplementary Fig. 1G). Therefore, *Mir34a* is not required for pancreatic development, and its absence does not result in compensatory upregulation of *Mir34b* or *Mir34c*.

To study the role of *Mir34a* in pancreatic cancer development we crossed the *Mir34a*^*fl/fl*^ mice with the well described Kras mouse model for PDAC^38^ to generate *Ptf1a*^*+/Cre*^*; Kras*^*+/LSL-G12D*^*; Mir34a*^*fl/fl*^ mice (hereafter called: *Kras*^*G12D*^*; Mir34a*^*Δ/Δ*^), which were analysed at specific time points.

**Figure 1:**
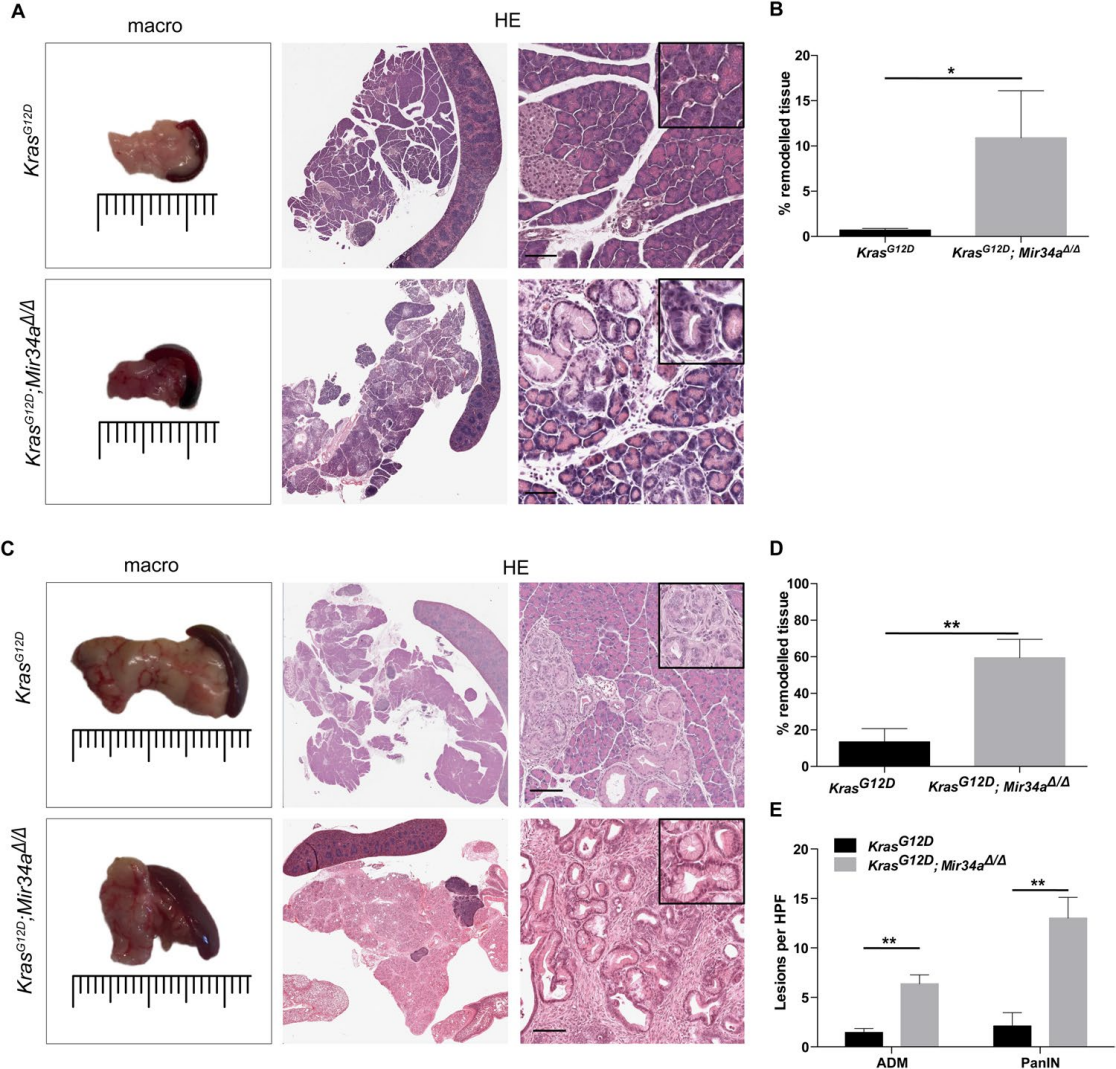
*Mir34a* knockout accelerates the development of ADM and PanIN lesions from a young age. (**A**) Macroscopic view of the pancreas of *Kras*^*G12D*^*; Mir34a*^*Δ/Δ*^ mice compared to *Kras*^*G12D*^ controls and haematoxylin eosin (HE) staining on whole slide and a 20 × zoomed in area, at 1 month of age. Scale bar 50 µm. (**B**) Quantification of the amount of pancreatic tissue remodelled (with ADM and PanIN lesions), expressed as percentage, in *Kras*^*G12D*^*; Mir34a*^*Δ/Δ*^ mice compared to *Kras*^*G12D*^ controls at 1 month of age (N ≥ 3 per group). (**C**) Macroscopic picture and HE staining of the pancreas from *Kras*^*G12D*^*; Mir34a*^*Δ/Δ*^ mice compared to *Kras*^*G12D*^ controls at 3 months of age. Scale bar 50 µm. (**D**) Quantification of the area of pancreatic tissue remodelled shown in percentage, in *Kras*^*G12D*^*; Mir34a*^*Δ/Δ*^ mice compared to *Kras*^*G12D*^ controls at 3 months of age (N ≥ 7 per group). (**E**) Quantification of the amount of ADM and PanIN lesions per high power field (HPF) in *Kras*^*G12D*^*; Mir34a*^*Δ/Δ*^ mice compared to *Kras*^*G12D*^ controls (N ≥ 7 per group).

At 1 month of age, the body weight and pancreas to body weight ratio were not significantly different in *Kras*^*G12D*^*; Mir34a*^*Δ/Δ*^ mice compared to controls (Supplementary Fig. 2A,B). Macroscopically, no difference between the pancreas from the two groups was observed (Fig. 1A). However, histological analysis revealed that *Kras*^*G12D*^*; Mir34a*^*Δ/Δ*^ mice already presented lesions (Fig. 1A). The area of remodelled tissue (containing ADM and PanIN lesions) was quantified, and as seen by HE analysis, *Kras*^*G12D*^*; Mir34a*^*Δ/Δ*^ mice presented a significantly higher percentage of remodelled tissue (Fig. 1B). Interestingly, this early remodelled phenotype was conserved at 3 months of age (Fig. 1C,D) and *Kras*^*G12D*^*; Mir34a*^*Δ/Δ*^ mice presented macroscopically a more fibrotic pancreas (Fig. 1C). These findings were confirmed by CK19 and MUC5AC staining as markers for ADM and PanIN lesions, respectively (Fig. 1E and Supplementary Fig. 2C). The ADM and PanIN lesions from *Kras*^*G12D*^*; Mir34a*^*Δ/Δ*^ mice were not more proliferative (Supplementary Fig. 2D,E) nor apoptotic (Supplementary Fig. 2D,F) compared to those from the *Kras*^*G12*^ control mice. Overall, these results show that *Kras*^*G12D*^*; Mir34a*^*Δ/Δ*^ mice present an acceleration in the development of ADM and PanIN pancreatic lesions, compared to *Kras*^*G12D*^ mice.

### Preneoplastic lesions of *Kras*^*G12D*^*; Mir34a*^*Δ/Δ*^ mice progress to invasive carcinomas at 6 months of age

**Figure 2:**
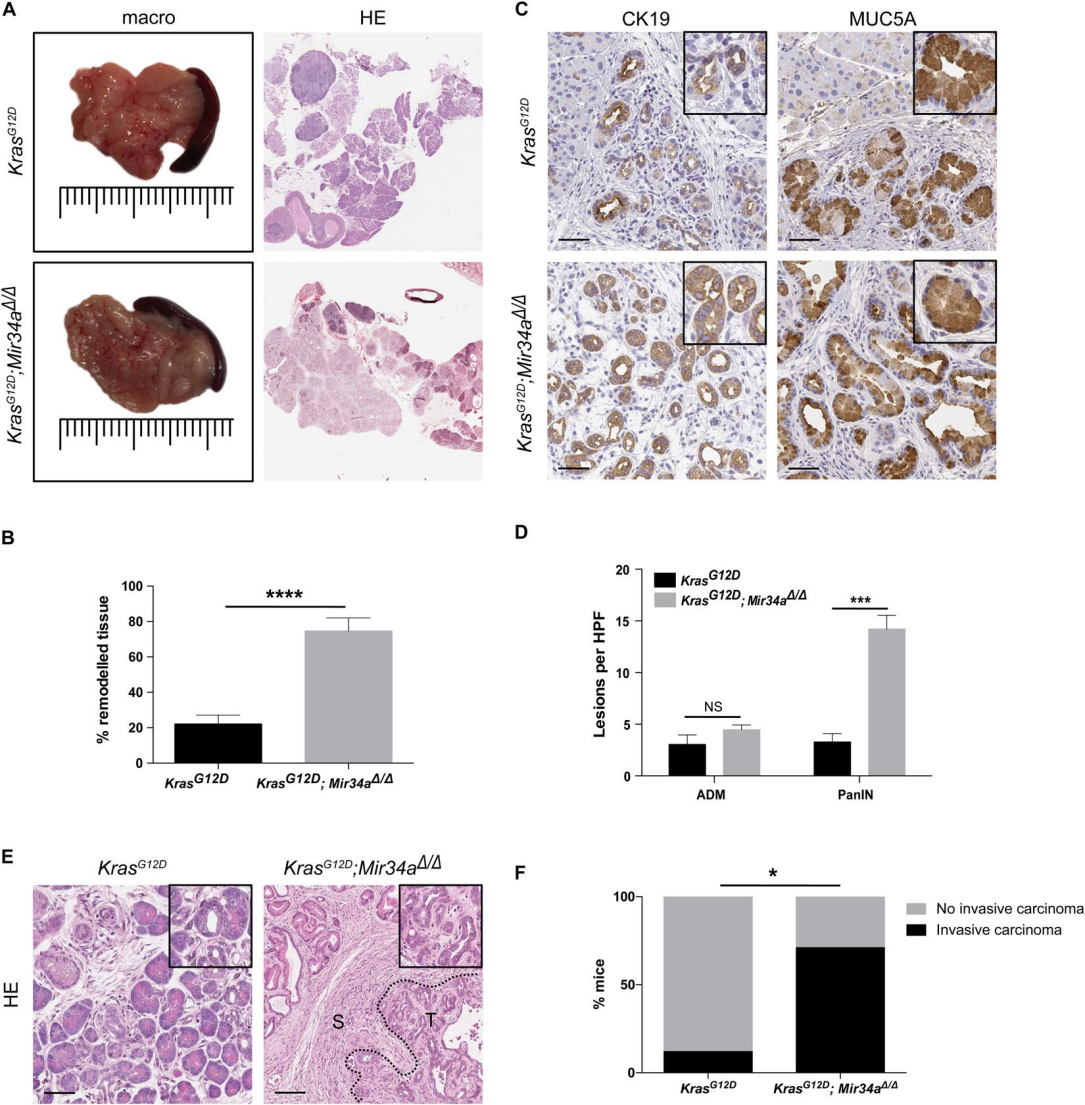
*Mir34a* knockout mice present invasive carcinomas at 6 months of age. (**A**) Macroscopic view of the pancreas of *Kras*^*G12D*^*; Mir34a*^*Δ/Δ*^ mice compared to *Kras*^*G12D*^ controls, HE staining of whole slide and a 20x zoomed in area at 6 months of age. (**B**) Quantification of the area, shown in percentage, of pancreatic tissue remodelled in *Kras*^*G12D*^*; Mir34a*^*Δ/Δ*^ mice compared to *Kras*^*G12D*^ controls. (N ≥ 8 per group). (**C**) Immunohistochemistry staining of the ductal marker CK19 and the PanIN marker MUC5A in *Kras*^*G12D*^*; Mir34a*^*Δ/Δ*^ mice compared to *Kras*^*G12D*^ controls. (**D**) Quantification of ADM and PanIN lesions at 6 months of age in *Kras*^*G12D*^*; Mir34a*^*Δ/Δ*^ mice compared to *Kras*^*G12D*^ controls. (N ≥ 8 per group). (**E**) HE staining showing an area of ADM and PanIN lesions in *Kras*^*G12D*^ mice compared to an area of PanIN lesions, stroma (S) and tumour (T) in *Kras*^*G12D*^*; Mir34a*^*Δ/Δ*^ mice. (**F**) Quantification of presence of invasive carcinomas, shown in percentage, in *Kras*^*G12D*^*; Mir34a*^*Δ/Δ*^ mice compared to *Kras*^*G12D*^ controls. Fisher’s test (N = 22). (**A,C,E**). Scale bar 50 µm.

To gain more information about the acceleration phenotype, mice at 6 months of age were analysed. Macroscopically, the pancreas of *Kras*^*G12D*^*; Mir34a*^*Δ/Δ*^ mice often presented areas with a hard mass, in comparison to that of *Kras*^*G12D*^ mice which did not show macroscopic tumours (Fig. 2A). These differences were also observed histologically, the pancreas of *Kras*^*G12D*^*; Mir34a*^*Δ/Δ*^ mice was very fibrotic and almost lacked normal exocrine areas (Fig. 2A). Furthermore, the pancreas of *Kras*^*G12D*^*; Mir34a*^*Δ/Δ*^ mice presented significantly larger remodelled areas (Fig. 2B) with slightly more CK19 positive ADMs and significantly more MUC5A positive PanIN lesions (Fig. 2C,D). Of note, *Kras*^*G12D*^*; Mir34a*^*Δ/Δ*^ mice already presented significantly more invasive carcinoma (including areas with microscopic carcinomas) in comparison to *Kras*^*G12D*^ controls (Fig. 2E,F). Therefore, these results show that the preneoplastic lesions observed in *Kras*^*G12D*^*; Mir34a*^*Δ/Δ*^ mice at an early age are aggressive and can quickly develop into invasive carcinomas already at 6 months of age.

**Figure 3:**
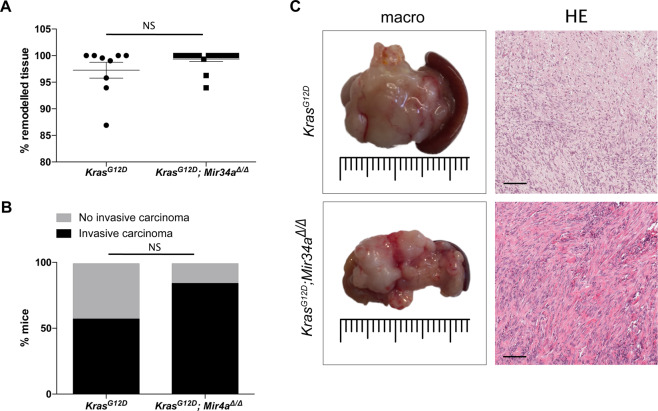
*Mir34a* knockout mice present more invasive carcinomas than *Kras*^*G12D*^ mice at terminal stage. (**A**) Percentage of pancreatic tissue remodelling from *Kras*^*G12D*^*; Mir34a*^*Δ/Δ*^ mice compared to *Kras*^*G12D*^ controls at terminal stage (N ≥ 9 per group). (**B**) Quantification of presence of invasive carcinomas in *Kras*^*G12D*^*; Mir34a*^*Δ/Δ*^ mice compared to *Kras*^*G12D*^ controls, shown in percentage. Fisher’s test (N = 33) OR4 (0.81,16.87). (**C**) Macroscopic picture of the invasive carcinoma from *Kras*^*G12D*^*; Mir34a*^*Δ/Δ*^ mice compared to that of *Kras*^*G12D*^ controls and HE staining of the invasive carcinoma area. Scale bar 50 µm.

At terminal stage the majority of *Kras*^*G12D*^*; Mir34a*^*Δ/Δ*^ mice presented a fully remodelled pancreas (Fig. 3A) but did not developed significantly more carcinomas than *Kras*^*G12D*^ mice (Fig. 3B). The lack of difference of invasive carcinoma was in agreement with the lack of increased metastasis to the lung and liver in *Kras*^*G12D*^*; Mir34a*^*Δ/Δ*^ (Supplementary Fig. 3A). In line with these results, the tumours of the two genotypes were macroscopically and histologically indistinguishable (Fig. 3C). Survival analysis between both groups presented a trend towards lower survival in *Kras*^*G12D*^*; Mir34a*^*Δ/Δ*^ mice (Supplementary Fig. 3B) supporting the acceleration phenotype hypothesis; however, the difference was not significant. Overall, these results show that the acceleration in lesion formation in *Kras*^*G12D*^*; Mir34a*^*Δ/Δ*^ mice results in higher tumour penetrance already at 6 months of age, and in a trend towards lower survival without affecting the metastatic potential.

### *Mir34a* ablation leads to a cell-autonomous activation of inflammatory pathways

**Figure 4:**
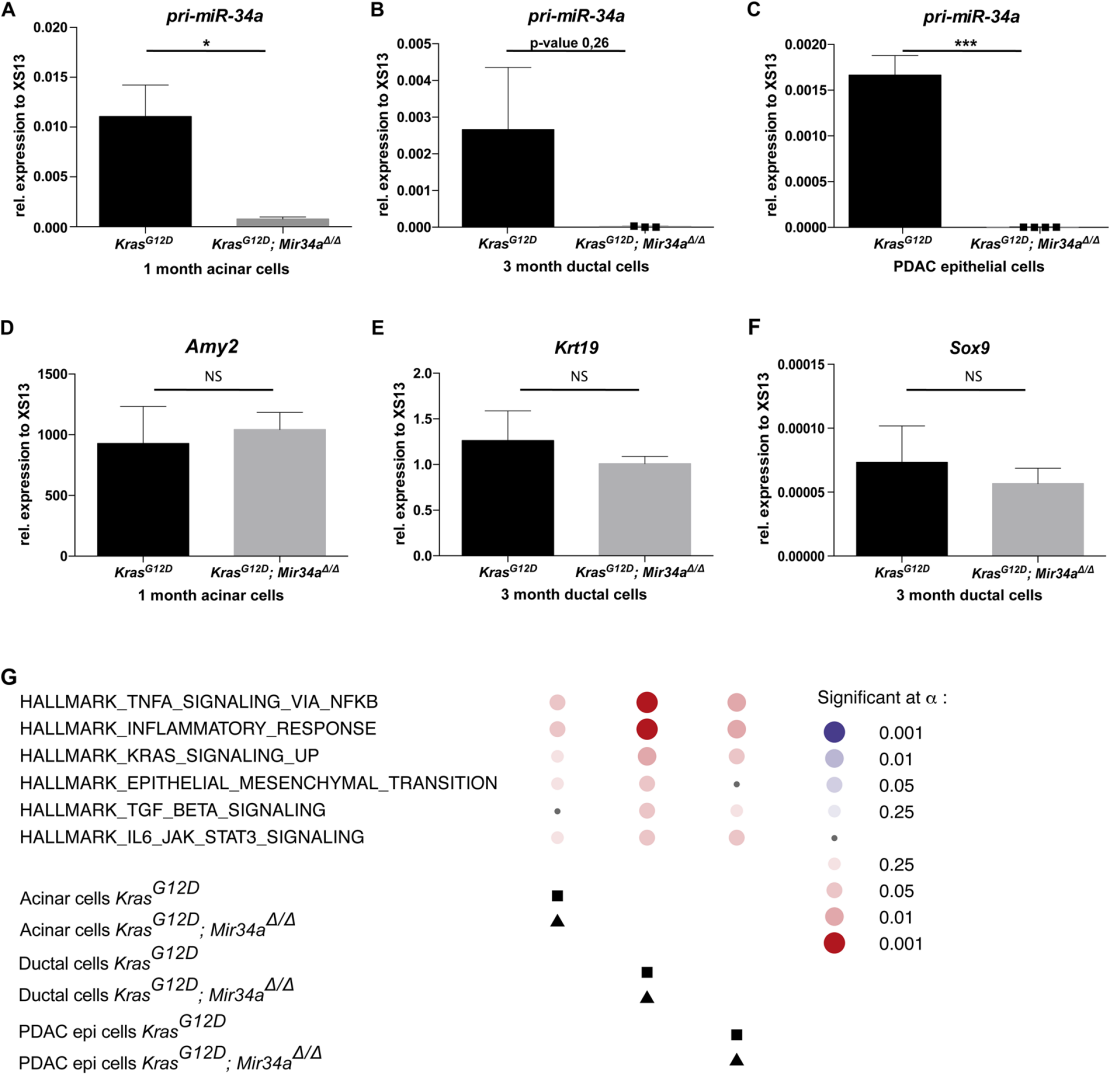
Upregulation of key signalling pathways in *Kras*^*G12D*^*; Mir34a*^*Δ/Δ*^ mice compared to *Kras*^*G12D*^ controls. (**A**) RNA expression of *pri-Mir-34a* in acinar explants at day 0 after isolation from *Kras*^*G12D*^*; Mir34a*^*Δ/Δ*^ mice and *Kras*^*G12D*^ controls at 1 month of age. Welch’s t-test (N = 4 per group). (**B**) RNA expression of *pri-Mir-34a* in ductal cells isolated from *Kras*^*G12D*^*; Mir34a*^*Δ/Δ*^ mice compared to *Kras*^*G12D*^ controls at 3 months of age. Welch’s t-test (N = 3 per group). (**C**) Expression of *pri-miR34a* in cell lines isolated from tumour tissue of *Kras*^*G12D*^*; Mir34a*^*Δ/Δ*^ mice compared to *Kras*^*G12D*^ controls at terminal stage. Welch’s t-test (N ≥ 4 per group). (**D**) RNA expression of the acinar marker Amylase in acinar explants at day 0 after isolation from *Kras*^*G12D*^*; Mir34a*^*Δ/Δ*^ mice and *Kras*^*G12D*^ controls at 1 month of age. Welch’s t-test (N = 4 per group). Differences are not statistically significant. (**E,F**) RNA expression of the ductal marker CK19 (**E**) and SOX9 (**F**) in ductal cells isolated from *Kras*^*G12D*^*; Mir34a*^*Δ/Δ*^ mice compared to *Kras*^*G12D*^ controls at 3 months of age. Welch’s t-test (N = 3 per group). Differences are not significant. (**G**) Bubble plot showing the top 6 pathways enriched in *Kras*^*G12D*^*; Mir34a*^*Δ/Δ*^ mice compared to *Kras*^*G12D*^ controls after performing gene set enrichment analysis in RNA extracted from acinar cell explants at 1 month of age, ductal cells at 3 months of age, and at tumour cells at terminal stage. Red circles show upregulated pathways and blue downregulated. The size of the circle represents the p-value. Squares represent the reference group (*Kras*^*G12D*^ mice), and triangles the treatment group (*Kras*^*G12D*^*; Mir34a*^*Δ/Δ*^ mice).

In order to gain insights into the mechanism of accelerated tumour formation and to avoid heterogeneous results due to the significant differences between the areas of normal exocrine tissue and those with pancreatic remodelling, specific cell types of *Kras*^*G12D*^*; Mir34a*^*Δ/Δ*^ and *Kras*^*G12*^ mice at each time point were isolated. Acinar cells were isolated from pancreata of 1 month old mice (since *Kras*^*G12D*^*; Mir34a*^*Δ/Δ*^ still present areas of normal exocrine tissue), ductal cells from remodelled pancreata of 6-month-old mice, and epithelial tumour cells were isolated from the invasive carcinomas of terminal mice. The purity of the isolated cells was assessed by gene expression analysis. All cells derived from *Kras*^*G12D*^*; Mir34a*^*Δ/Δ*^ mice did not express *Mir34a* (as shown by absence of pri-Mir34a mRNA expression) compared to *Kras*^*G12D*^ controls (Fig. 4A–C). Acinar cells from both *Kras*^*G12D*^*; Mir34a*^*Δ/Δ*^ and *Kras*^*G12*^ mice, expressed comparable mRNA levels of *Amylase* (Fig. 4D) and did not express mRNA from the ductal marker *CK19* (data not shown). In contrast, the ductal cells from both *Kras*^*G12D*^*; Mir34a*^*Δ/Δ*^ and *Kras*^*G12*^ mice expressed comparable mRNA levels of the ductal markers *CK19* (Fig. 4E) and *SOX9* (Fig. 4F) and they did not express mRNA from the acinar marker *Amylase* (data not shown). Therefore, the same subtype of cells was isolated in both genotypes.

In a second step, RNA sequencing and gene set enrichment analysis (GSEA) of the aforementioned cell types were performed. RNA from *Kras*^*G12D*^*; Mir34a*^*Δ/Δ*^ mice showed an enrichment in the following pathways: TNFA via NFKB, inflammatory response, Kras signalling up, EMT, TGFB, IL6-JAK-STAT3, across cell types compared to that of *Kras*^*G12*^ controls (Fig. 4G and Supplementary Table 1). This result suggests that acinar cells generate an autocrine inflammatory response. Furthermore, this result supports the lesion acceleration hypothesis by showing an enrichment in Kras signalling already in ductal cells at 3 months of age.

**Figure 5:**
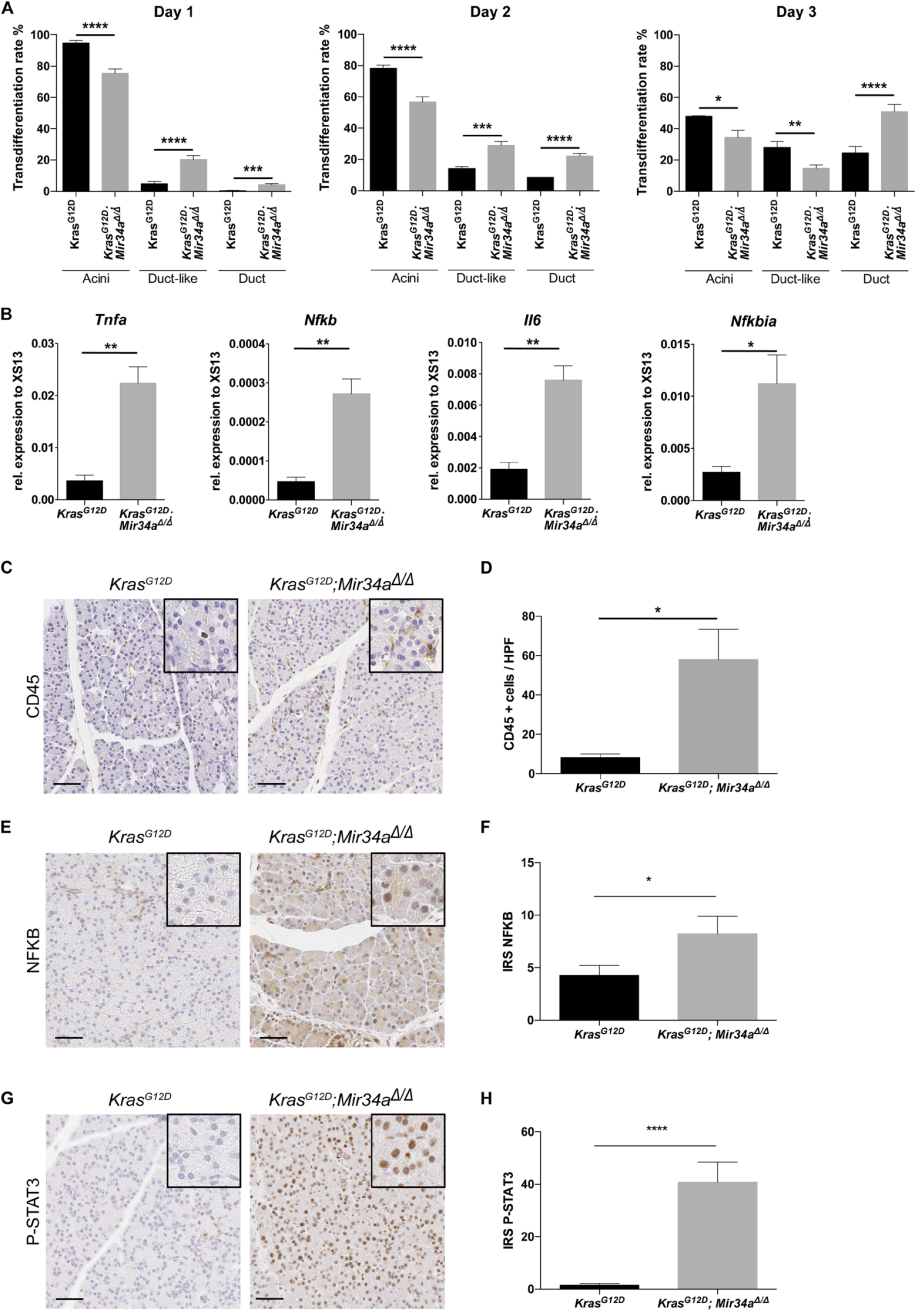
*Mir34a* knock out results in an inflammatory phenotype. (**A**) Average of the transdifferentiation rate of acinar cell explants from pancreata of *Kras*^*G12D*^*; Mir34a*^*Δ/Δ*^ and *Kras*^*G12D*^ mice into acini, duct-like and duct structures (N ≥ 3 per group). Unpaired Student’s t-test with combined SD. (**B**) RNA expression of members of the NFKB signalling pathway: *Tnfa*, *Nfkb*, *Il6*, and *Nfkbia*, in acinar cell explants directly after isolation (day 0) from *Kras*^*G12D*^*; Mir34a*^*Δ/Δ*^ mice compared to *Kras*^*G12D*^ controls. Welch’s t-test (N = 4 per group). (**C**) Immunohistochemistry staining for CD45 in tissue from *Kras*^*G12D*^*; Mir34a*^*Δ/Δ*^ mice compared to *Kras*^*G12D*^ controls. Scale bar 50 µm. (**D**) Quantification of CD45 positive cells per high power field in areas of normal tissue from *Kras*^*G12D*^*; Mir34a*^*Δ/Δ*^ mice compared to *Kras*^*G12D*^ controls (N ≥ 3 per group). (**E**) Immunohistochemistry staining for NFKB in tissue from *Kras*^*G12D*^*; Mir34a*^*Δ/Δ*^ mice compared to *Kras*^*G12D*^ controls. Scale bar 50 µm. (**F**) Quantification of NFKB positive nuclei from acinar cells in areas of normal tissue per high power field from *Kras*^*G12D*^*; Mir34a*^*Δ/Δ*^ mice compared to *Kras*^*G12D*^ controls (N ≥ 3 per group). (**G**) Immunohistochemistry staining for P-STAT3 in tissue from *Kras*^*G12D*^*; Mir34a*^*Δ/Δ*^ mice compared to *Kras*^*G12D*^ controls. Scale bar 50 µm. (**H**) Quantification of P-STAT3 positive nuclei from acinar cells in areas of normal tissue per high power field from *Kras*^*G12D*^*; Mir34a*^*Δ/Δ*^ mice compared to *Kras*^*G12D*^ controls (N ≥ 3 per group).

To confirm our hypothesis of a faster lesion development in the *Kras*^*G12D*^*; Mir34a*^*Δ/Δ*^ mice, acinar cell explants from 1-month old mice were isolated, cultured and their transdifferentiation into ductal cells was followed for 3 days (Fig. 5A). Acinar cells from *Kras*^*G12D*^*; Mir34a*^*Δ/Δ*^ mice showed accelerated ADM transdifferentiation rates *in vitro* (Fig. 5A and Supplementary Fig. 4B). Therefore, acini from *Kras*^*G12D*^*; Mir34a*^*Δ/Δ*^ mice transdifferentiate into ductal structures, faster than *Kras*^*G12*^ controls, in a cell-autonomous manner.

Next, the mRNA expression levels of members of the signalling pathways enriched by GSEA were analysed in acinar cell explants. RNAs of freshly isolated acinar cell explants were analysed; expression of *Tnfa*, *Nfkb*, *Il6* and *Nfkbia* was significantly upregulated in *Kras*^*G12D*^*; Mir34a*^*Δ/Δ*^ acinar cell explants (Fig. 5B). This result validates the GSEA results observed after sequencing. In summary, our results suggest that acinar cells of *Kras*^*G12D*^*; Mir34a*^*Δ/Δ*^ mice develop to ADM in a cell-autonomous manner and they secrete factors which attract inflammatory cells, possibly accounting for the acceleration of invasive carcinoma development.

To confirm this hypothesis, the presence of inflammatory cells in the non-remodelled pancreas at 1 month of age was analysed (Fig. 5C). There were significantly more CD45 positive cells in *Kras*^*G12D*^*; Mir34a*^*Δ/Δ*^ mice compared to *Kras*^*G12*^ controls (Fig. 5D). In addition, we validated an active TNFA and IL6 signalling in acinar cells by immunohistochemical staining of NFKB and P-STAT3 (Fig. 5E,G, respectively). The acinar cells from *Kras*^*G12D*^*; Mir34a*^*Δ/Δ*^ mice had a slightly increased NFKB expression and significantly more P-STAT3 expression compared to *Kras*^*G12*^ controls (Fig. 5E–H). These results demonstrate that in *Kras*^*G12D*^*; Mir34a*^*Δ/Δ*^ mice TNFA and IL6 expression is increased in the acinar compartment already at the age of 1 month leading to an inflammatory response and recruitment of inflammatory cells to the tissue.

## Discussion

Many studies showed that plenty of microRNAs (miRNAs) are deregulated in PDAC, among them *Mir34a* is often downregulated and is a promising biomarker with prognostic value that correlates with diagnosis^39–41^. However, the exact mechanism by which *Mir34a* exerts its tumour suppressor role in PDAC is not clear yet.

Here we present an *in vivo* study where we investigated the role of *Mir34a* in PDAC carcinogenesis using genetically engineered mouse models. Conditional deletion of *Mir34a* in the pancreas of mice led to a significant acceleration in the formation of pancreatic pre-neoplastic ADM and PanINs lesions already at the age of 1 month. Furthermore, this also accelerated the formation of pancreatic invasive carcinomas, which were present already at 6 months of age. These results resemble the data from human PDAC patients in which patients with low *Mir34a* expression present a lower survival rate^30,32–34^. Furthermore, in agreement with our histological observations, acinar explants isolated from the pancreas of 1-month-old mice transdifferentiated faster into ductal structures in culture, suggesting a cell-autonomous mechanism. We hypothesized that the reason is that *Mir34a* ablation in acini from *Kras*^*G12D*^*; Mir34a*^*Δ/Δ*^ mice results in an alteration of signalling pathways promoting acinar differentiation. As shown by RNA sequencing and gene set enrichment analysis (GSEA), EMT, TGFB, IL6-JAK-STAT3, TNFA via NFKB, Kras signalling up, and inflammation pathways are enriched in *Kras*^*G12D*^*; Mir34a*^*Δ/Δ*^ mice. Additionally, the expression of *Tnfa*, *Nfkb* and *Il6* is significantly increased, there are significantly more CD45 positive immune cells in areas of normal exocrine tissue, and significantly more NFKB and P-STAT3 accumulates in the nucleus of normal acinar cells. Based on all of these results, and since persistent inflammation in a Kras^G12D^ setting results in PDAC^42^, it is plausible that depletion of *Mir34a* expression drives an upregulation of the inflammatory cytokines, specifically in acinar cells. This leads to NFKB and P-STAT3 activation (favouring pre-neoplastic lesion development and carcinogenesis) and to the recruitment of inflammatory cells which eventually accelerate PDAC development. In line with these findings, *Mir34a* was shown to negatively regulate the IL6R/STAT3 pathway in sporadic and colitis-associated colorectal cancer and thereby contribute to invasion and metastasis^37,43^.

MicroRNAs (mRNAs), including *Mir34a*, were reported to be key inflammation regulators (reviewed in^44^). The signalling pathways enriched by GSEA in *Kras*^*G12D*^*; Mir34a*^*Δ/Δ*^ mice, are inflammatory pathways strongly related between themselves and their upregulation was in line with the literature. One of the signalling pathways enriched at early time points was TGFB. A recent study with mouse models, revealed that activation of TGFB pathway during early pancreatic tumorigenesis induces ADM reprogramming, by activating apoptosis and cell differentiation and provides a favourable environment for the development of Kras^G12D^-driven preneoplastic lesions and carcinogenesis^45^. This was also confirmed in human pancreatic cells^46,47^. Furthermore, SMAD4, the main effector of the TGFB signalling pathway is also a direct target of *Mir34a*^48–50^. Additionally, IL6-JAK-STAT3, TNFA via NFKB and inflammation pathways are also enriched by GSEA and we show that ablation of *Mir34a* in *Kras*^*G12D*^*; Mir34a*^*Δ/Δ*^ mice led to an increase in TNFA and IL6 in pancreatic acini. Multiple studies using mouse models of pancreatic cancer demonstrate a strong relationship between Kras, NFKB, STAT3 and cytokine signalling which drives lesion formation and the development of PDAC^51–57^.

Our results are in agreement with previous studies showing that *Mir34a* downregulates TNFA and IL6^58^. For instance, *in vitro* administration of *Mir34a* mimics to LPS treated macrophages decreases the expression of TNFA and IL6, and reduces NFKB activation. Here the mechanism shown is direct inhibition of *Mir34a* over either its target Notch1, which activates the inflammatory response in macrophages, or other genes that affect NFKB signalling; therefore, modulating LPS-induced macrophage inflammatory response^58^. Furthermore, in colorectal cancer *Mir34a* was shown to constrain carcinogenesis by directly inhibiting an IL-6R/STAT3/*Mir34a* feedback loop, and its ablation is required to induce IL6-mediated EMT and invasion^37^. Moreover, a couple of studies support that IL-6R is a direct target of *Mir34a*^43,59,60^. Therefore, many previous studies have reported similar results to ours in other organs and supports our observation that *Mir34a* ablation results in the upregulation of the inflammatory cytokines TNFA and IL6 in the pancreas. Interestingly, these two secreted pro-inflammatory cytokines are considered strong EMT inducers key for cancer progression^61^. However, in our study despite that an enrichment in EMT signature was found at early time points, it was not sufficient to result in significantly more metastasis in *Kras*^*G12D*^*; Mir34a*^*Δ/Δ*^ mice, possibly due to the fact that at later stages of the disease *Kras*^*G12*^ mice also present a strong EMT signature or due to the faster tumour development.

In summary, this study shows that ablation of *Mir34a* results in a cell-autonomous inflammatory response marked by the increase in TNFA and IL6 expression in acinar cells, which is linked to an enhanced TGFB signalling in preneoplastic transformation that appears to accelerate *Kras*^*G12D*^-dependent pancreatic carcinogenesis. Overall, our results suggest that apart from its known tumor suppressor role, *Mir34a* also has an anti-inflammatory role in the pancreas by downregulating TNFA and IL6. This anti-inflammatory role may be important at the initiation stage of preneoplastic development, but further functional understanding of the underlying mechanisms is required in order to fully support this hypothesis. This capacity of *Mir34a* should also be taken into account when designing *Mir34a* targeted therapy for PDAC.

## Methods

### Mouse strains

*Mir34a*^*fl/fl*^ mice^37^ were generated by the Hermeking laboratory and we bred them to *Ptf1a*^*+/Cre*^ ^62^ and *Kras*^*+/LSL-G12D*^ ^38^ mice to generate *Ptf1a*^*+/Cre*^*; Mir34a*^*fl/fl*^ (called: *Mir34a*^*Δ/Δ*^) and *Ptf1a*^*+/Cre*^*; Kras*^*+/LSL-G12D*^*; Mir34a*^*fl/fl*^ (called: *Kras*^*G12D*^*; Mir34a*^*Δ/Δ*^). Co-housed wild type *Mir34a*^*fl/*fl^ and *Ptf1a*^*+/Cre*^*; Kras*^*+/LSL-G12D*^ (called: *Kras*^*G12D*^) littermate mice were used as controls. All animal experiments were conducted in accordance with German Federal Animal Protection Laws and approved by the Institutional Animal Care and Use Committee from the Technical University of Munich (Germany), and from the Government of Bavaria AZ: (5.2-1-54-2532-46-2014).

### Body and pancreatic weight analysis

Mice were sacrificed and immediately weighted in a scale. Subsequently, the whole pancreas was carefully excised without any adjacent tissue and directly weighed in a precision scale under sterile technique.

### Primary cell isolation and culture

After sacrificing the mice, either acinar, ductal or tumour cells (from terminal mice) were directly isolated from the pancreas and cultured using the methods previously described^63,64^. For quantification of acinar explants transdifferentiation rate, acinar cell explants were counted in 6 to 33 high power fields per mouse using a 10X objective (approx. 3 areas per well of a 48 well plate). The phenotype was defined as acinar (cluster of acinar cells resembling a ball), duct-like (acinar cell clusters with a ductal structure emerging within the cluster) or ductal (ductal structure with a defined lumen), see Supplementary Fig. 4A.

### Immunohistochemical staining

Immunohistochemistry was performed as previously described^65^. The following primary antibodies were used: rabbit anti-Cleaved caspase 3 (1:200; Cell Signalling #9661), mouse anti-CD45 (1:20, BDPharmigen #550539), rabbit anti-CK19 (1:1000; Abcam #ab133496), goat anti-CPA1 (1:300, RD Systems #AF2765), mouse anti–Ki-67 (1:400; BDPharmigen #550609), mouse anti-MUC5AC (1:200; Cell Marque #292M-95), rabbit anti-p65 (C-20) (1:200, Santa Cruz #sc-372), rabbit anti-phospho-STAT3 (Y705) (1:100, Cell Signaling #9145).

### Morphometric quantification

After staining, slides were scanned at 20x using a Leica AT2 scanner (Leica) and analysed with Aperio Image Scope program (Leica). Whole pancreatic area and remodelled tissue (areas of ADM and/or low/high grade PanIN lesions) were quantified in a blinded way; representative images were taken. For quantification of CK19 positive ADM and MUC5AC-positive PanIN lesions, 10 high power fields (HPF) were counted per whole pancreatic tissue slide and the average number of lesions per HPF was calculated. For quantification of Ki-67 and cleaved caspase 3, 50 ADM and PanIN lesions were randomly selected across the whole slide (no more than 10 lesions in the same area), positive cells (visualized by brown precipitate) were counted and the percentage was calculated. The total number of cells counted per HPF excluded: fatty, edema, or inflammatory tissue. Presence of microscopic carcinomas (only a few cancer cells) and pancreatic ductal adenocarcinoma was determined by an experienced pathologist (K.S.). For the quantification of CD45 positive immune cells, 10 HPF were randomly selected across the whole pancreatic tissue slide in areas of normal tissue. Only positive immune cells were counted. For the quantification of NFKB and P-STAT3 positive nuclei the nuclear counting algorithm V9 from Image Scope was used with the following settings: threshold for cytoplasmic correction 230, upper limit of weak (1+) 217, moderate (2+) 200 and strong (3+) 188. 10 HPF were randomly selected across the whole pancreatic tissue section in areas of normal tissue. Only nuclei from normal acini were counted.

### RNA isolation and quantitative RT-PCR

After sacrificing the mice, a small piece of the pancreas was excised, stored in RNAlater (Qiagen) overnight at 4 °C and stored at −80 °C the next morning. RNA was homogenized with RA1 lysis buffer (Macherey-Nagel) and β-mercaptoethanol. Subsequently, RNA was isolated using the Maxwell 16 LEV simplyRNA Purification Kit (Promega) following manufacturer’s instructions. SuperScript II Reverse Transcriptase (Invitrogen) was used for cDNA synthesis, according to manufacturer’s protocol. RT-PCR was performed as previously described^65^ using the SYBR Green master mix (Roche) on a Lightcycler480 system (Roche). Primers used are described in Table 1. Melting curve analysis was performed to ensure product quality and specificity. Expression levels of each transcript were normalized to the housekeeping gene XS13 (a constitutively expressed ribosomal protein with same levels in normal, cancerous, and inflamed human pancreas)^66^, using the ΔΔCt method. All RT-PCR experiments were performed with at least N = 3 individual biological samples per group.

**Table 1:** Primers used for RT-PCR.

Gene	Primer	Sequence (5′-3′)
*Mir34a*	*Pri-miR-34a_*F	5′-CTGTGCCCTCTTGCAAAAGG-3′
	*Pri-miR-34a_*R	5′-GGACATTCAGGTGAGGGTCTTG-3′
*Mir34bc*	*Pri-miR-34bc_*F	5′-GGCAGGAAGGCTCCAGATG-3′
	*Pri-miR-34bc_*R	5′-CCTCACTGTTCATATGCCCATTC-3′
*Amylase*	*Amy2a*_F	5′-TGGTCAATGGTCAGCCTTTTTC-3′
	*Amy2a*_R	5′-CACAGTATGTGCCAGCAGGAAG-3′
*CK19*	*Krt19*_F	5′-ACCCTCCCGAGATTACAACC-3′
	*Krt19*_R	5′-CAAGGCGTGTTCTGTCTCAA-3′
*SOX9*	*SOX9*_F	5′-CCACGTGTGGATGTCGAAG-3′
	*SOX9*_R	5′-CTCAGCTGCTCCGTCTTGAT-3′
*Tnfa*	*Tnfa*_F	5′-TGCCTATGTCTCAGCCTCTTC-3′
	*Tnfa*_R	5′-GAGGCCATTTGGGAACTTCT-3′
*Nfkb*	*Nfkb*_F	5′-GGAGGCATGTTCGGTAGTGG-3′
	*Nfkb*_R	5′-CCCTGCGTTGGATTTCGTG-3′
*Il6*	*Il6*_F	5′-GCTACCAAACTGGATATAATCAGGA-3′
	*Il6*_R	5′-CCAGGTAGCTATGGTACTCCAGAA -3′
*Nfkbia*	*Nfkbia*_F	5′-TGAAGGACGAGGAGTACGAGC-3′
	*Nfkbia*_R	5′-TCTTCGTGGATGATTGCCAAG-3′
*XS13*	*XS13*_F	5′-TGGGCAAGAACACCATGATG-3′
	*XS13*_R	5′-AGTTTCTCCAGAGCTGGGTTGT-3′

### RNA-sequencing

Library preparation for bulk 3′-sequencing of poly(A)-RNA was done as described previously^67^. Briefly, barcoded cDNA of each sample was generated with a Maxima RT polymerase (Thermo Fisher) using oligo-dT primer containing barcodes, unique molecular identifiers (UMIs) and an adapter. 5′ ends of the cDNAs were extended by a template switch oligo (TSO) and after pooling of all samples full-length cDNA was amplified with primers binding to the TSO-site and the adapter. cDNA was tagmented with the Nextera XT kit (Illumina) and 3′-end-fragments finally amplified using primers with Illumina P5 and P7 overhangs. The library was sequenced on a NextSeq. 500 (Illumina) with 16 cycles for the barcodes and UMIs in read1 and 65 cycles for the cDNA in read2.

### RNAseq analysis

Gencode gene annotations version M18 and the mouse reference genome major release GRCm38 were derived from the Gencode homepage (https://www.gencodegenes.org/). Dropseq tools v1.12^68^ was used for mapping the raw sequencing data to the reference genome. The resulting UMI filtered countmatrix was imported into R v3.4.4. Prior differential expression analysis with DESeq. 2 1.18.1^69^, dispersion of the data was estimated with a parametric fit. The Wald test was used for determining differentially regulated genes between experimental conditions and shrunken log2 fold changes were calculated afterwards, with setting the type argument of the lfcShrink function to ‘normal’.

A gene was determined to be differentially regulated if the adjusted p-value was below 0.05. Gene set enrichment analysis was conducted with the preranked GSEA method^70^ within the MSigDB Hallmark database. Genes were ranked according to their respective log2 fold change. A pathway was considered to be significantly associated with an experimental condition at an alpha level of 0.05 (for NES and FDR values see Supplementary Table 1).

### Statistical analysis

Statistical analysis was performed using Graph Pad Prism6 (GraphPad Software Inc). Unless otherwise stated, the Mann-Whitney Test for non-normal distributed unpaired data was used for inter-group comparison. For Fischer’s test OR (95% CI) was used. Kaplan–Meier curve was calculated using the survival time for each mouse from the littermate groups. The log-rank test was used to address significant differences between the groups. For RT-PCR data, Log2 values were used for conducting the t-test. Welch’s t-test was used when samples followed a Gaussian distribution but they had different standard deviations. For all statistical analysis, differences with a p-value lower than 0.05 were considered significant, and the following scale was applied: *p < 0.05, **p < 0.01, ***p < 0.001, ****p < 0.0001. Data are presented as mean values ± SEM, unless otherwise stated.

## Supplementary information

Supplementary Information.
