# Supplementary material for: Mir34a constrains pancreatic carcinogenesis
Source: Sci Rep. 2020 Jun 15;10:9654. doi: 10.1038/s41598-020-66561-1 (PMC7295749; doi:10.1038/s41598-020-66561-1)
Supplement: Supplementary file 1 — Supplementary Information. [file 41598_2020_66561_MOESM1_ESM.pdf]

Mir34a constrains pancreatic carcinogenesis

Ana Hidalgo-Sastre[1], Clara Lubeseder-Martellato[1], Thomas Engleitner[2], Katja Steiger[3], Suyang Zhong[1], Judit Desztics[1], Rupert Öllinger[2], Roland Rad[2], Roland M. Schmid[1], Heiko Hermeking[4,5], Jens T. Siveke[6,7,8], and Guido von Figura[1*].

[1] Klinik und Poliklinik für Innere Medizin II, Technical University of Munich, Germany.

[2] Institute of Molecular Oncology and Functional Genomics, Department of Medicine II and TranslaTUM Cancer Center, Klinikum rechts der Isar, Technical University of Munich, Munich, Germany.

[3] Institute of Pathology, Technical University of Munich, Germany.

[4] Experimental and Molecular Pathology, Institute of Pathology, Ludwig Maximilian University Munich, Munich, Germany.

[5] German Cancer Consortium (DKTK), Partner site Munich, Germany

[6] Institute for Developmental Cancer Therapeutics, West German Cancer Center, University Hospital Essen, Essen, Germany

[7] Division of Solid Tumor Translational Oncology, German Cancer Consortium (DKTK, partner site Essen) and German Cancer Research Center, DKFZ, Heidelberg, Germany;

[8] German Cancer Consortium (DKTK), Partner site Essen, Germany

Supplementary Figures

Supplementary Figure 1

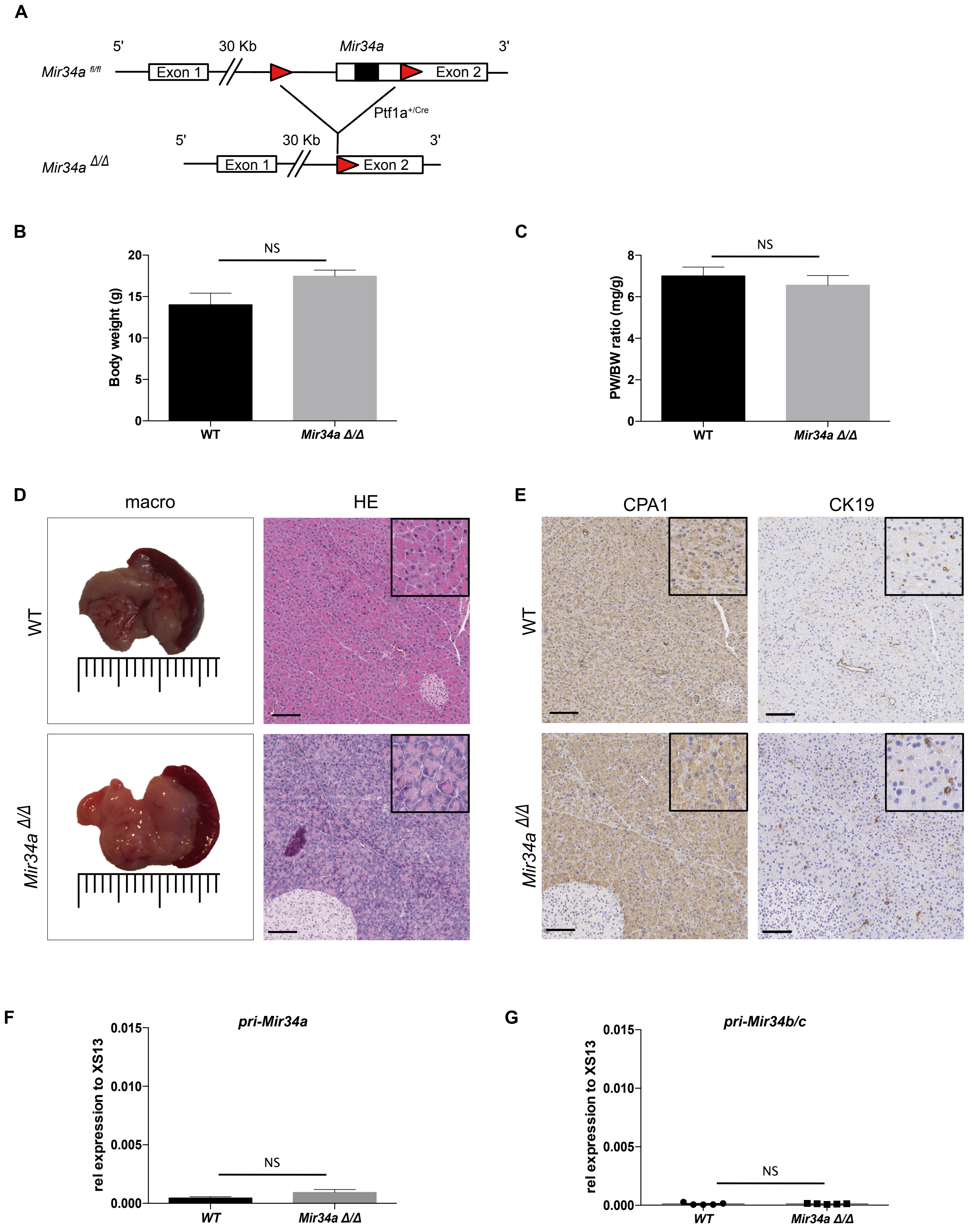

Supplementary Figure 2

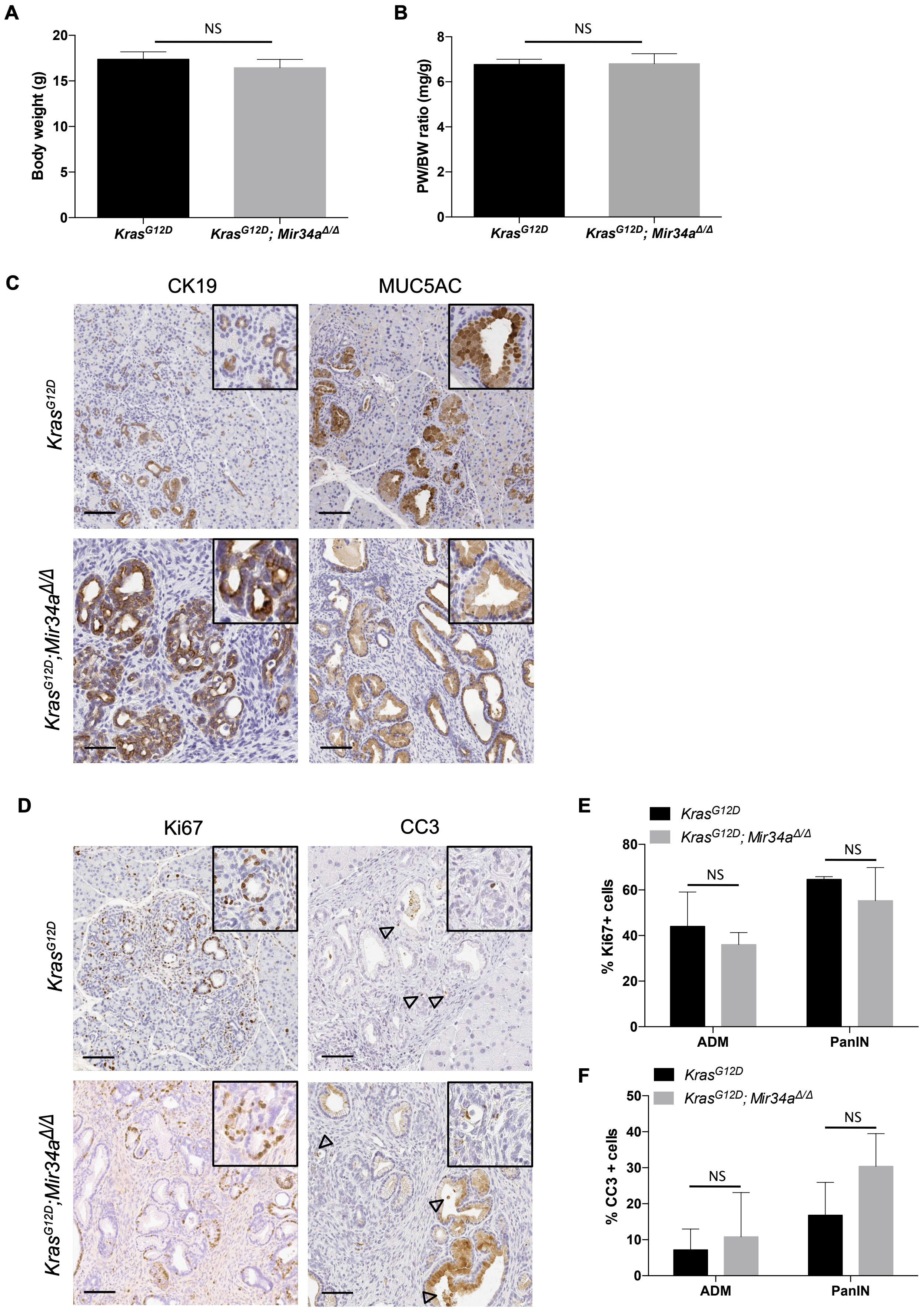

Supplementary Figure 3

A

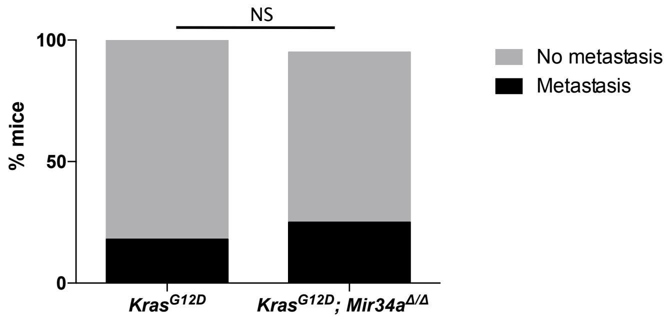

B

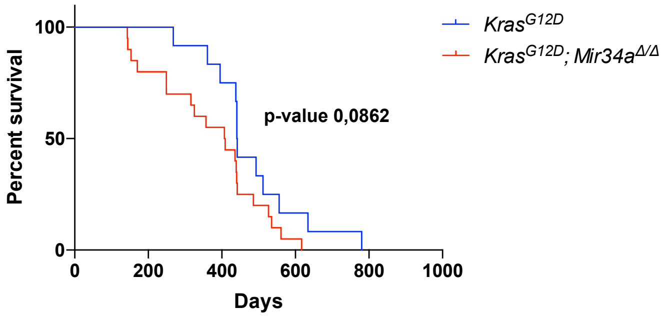

Supplementary Figure 4

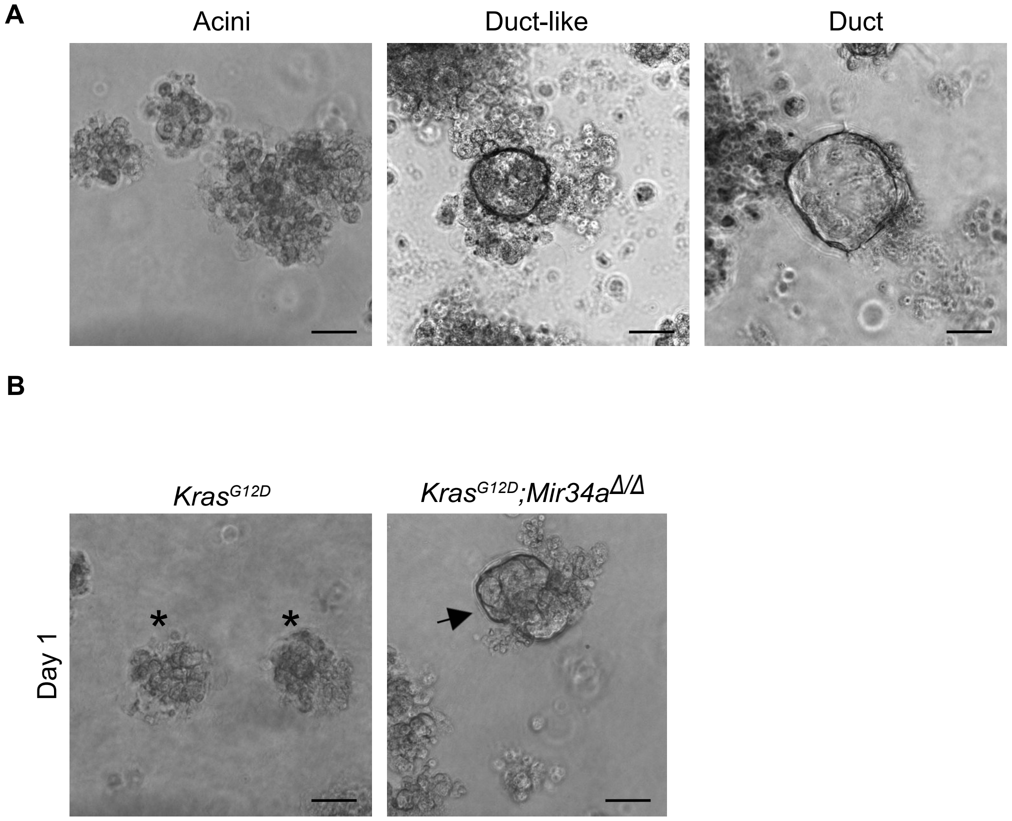

Supplementary Table 1. NES and FDR values for GSEA.

Pathways	Cell type	NES	FDR
HALLMARK_TNFA_SIGNALING_VIA_NFKB	Acini	1,80	0,02
	Ductal	0,0	0,0
	PDAC	1,96	0,002
HALLMARK_INFLAMMATORY_RESPONSE	Acini	1,69	0,02
	Ductal	0,0	9.986991E-4
	PDAC	1,92	0,001
HALLMARK_KRAS_SIGNALING_UP	Acini	1,25	0,15
	Ductal	0,0	0,003
	PDAC	1,53	0,03
HALLMARK_EPITHELIAL_MESENCHYMAL_TRANSITION	Acini	1,41	0,08
	Ductal	0.001	0,01
	PDAC	1,08	0,42
HALLMARK_TGF_BETA_SIGNALING	Acini	-1,02	0,86
	Ductal	0,02	0,02
	PDAC	1,43	0,06
HALLMARK_IL6_JAK_STAT3_SIGNALING	Acini	1,38	0,09
	Ductal	0,04	0,04
	PDAC	1,60	0,02
